# Abstracts From JADPRO Live at APSHO 2017

**Published:** 2018-01-01

**Marriott Marquis, Houston, Texas**

**November 2–5, 2017**

The posters associated with the abstracts below are available to view at eventscribe.com/2017/posters/JADPROLIVE/home.asp

## JL501. Advanced Practice Provider Delivery of Advance Care Planning Counseling in Community Oncology

Sabrina Mikan, PhD, RN, ACNS-BC, Supportive Care Programs, Texas Oncology, Austin, Texas

*Background:* Advanced practice providers (APPs) are vital in the team management of oncology. A key role of the APP is assisting patients and families with therapy review and coordination sessions where treatment questions and topics are discussed. Many topics are reviewed, rapport is developed, and the APP is able to introduce Advance Care Planning (ACP) concepts while assessing for any existing advance directives (ADs). ADs are important for the entire oncology care team to know about as care is delivered. When patients do not have ADs, through a systematic process beginning at their first visit together, the APP can introduce ACP basics and potentially refer interested patients for follow-up conversations.

*Methods:* APPs systematically introduce ACP at the therapy education session. Later, as early as cycle 3 of treatment, the APP re-introduces ACP and attempts a referral. Vital to ACP success is removing the stigma from when ACP is traditionally completed: during times of crisis. By including ACP in each patient’s plan of care, the opportunity is normalized and patients find it helpful to complete their ADs with the APP. Use of a Patient Values Assessment (PVA) is also effective to meet the patient’s goals of care.

*Results:* Over an 18-month timeframe beginning January 2016, ACP Counseling and Patient Values were measured across 60 practices offering the ACP program. At baseline month, ACP counseling was 64 and increased to 410 in June 2017; a 6-fold increase. Patient Values questionnaires at baseline were 25, increasing to 206 in June 2017; an 8-fold increase. Steady growth took place monthly along with increasing buy-in from each APP and practice leadership.

*Conclusions and Recommendations:* APPs each develop their personalized method for re-introducing ACP to their active treatment patients. With an identification process beginning at therapy teaching, the APP is able to periodically re-introduce ACP and attempt referrals for counseling. Utilizing specific, measureable, attainable, realistic, and timely (S.M.A.R.T.) goals created by each APP and the practice, there will be objective evaluation for increased improvement. Patient empowerment and education is at the heart of ACP. Monthly review of progress and continual quality improvement of the program process will help increase program access to every patient.

## JL502. An Algorithm for Transitioning Patients From Deferasirox Dispersible Tablets to Deferasirox Film-Coated Tablets and Sprinkle Granules

Lindsey M. Lyle, MS, PA-C, University of Colorado, Denver, Colorado, and Allyson Price, MS, PA-C, The University of Texas MD Anderson Cancer Center, Houston, Texas

*Context:* Iron chelation therapy for iron overload (IO) improves organ function and survival among patients. The deferasirox dispersible tablet (DT) has a well-defined safety and efficacy profile, yet patient adherence is not optimal secondary to gastrointestinal (GI) intolerability and palatability. This led to development of film-coated tablets (FCT) taken with or without a meal and sprinkle granules for sprinkling on soft food. Both contain the same active substance as DT but different excipients. In the ECLIPSE trial (2016), comparable FCT and DT safety profiles were demonstrated in patients with transfusion-dependent thalassemia or low-to-intermediate-risk myelodysplastic syndromes. Patients receiving FCT reported better compliance, continued treatment longer, and experienced greater serum ferritin (SF) reduction. Advanced practitioners (APs) need education regarding these new formulations and proper transition from the DT to them.

*Method:* An algorithm was developed based on the deferasirox prescribing information supplemented by practice recommendations. *Results:* Transitioning a patient from deferasirox DT to FCT is based on the patient’s response to deferasirox DT or other iron chelators, determined by SF and the degree of GI toxicity. Transitioning often begins at prescription renewal. Deferasirox FCT has greater bioavailability versus deferasirox DT; therefore, the recommended dose of FCT is ~30% lower than DT, rounded to the nearest whole tablet. Deferasirox FCT is available in 90, 180, and 360 mg tablets. The starting dose of FCT for treatment of chronic iron overload due to blood transfusions is 14 mg/kg/day (7 mg/kg/day for non–transfusion-dependent thalassemia [NTDT]). Monitoring for dose titration of deferasirox FCT follows the same schedules as DT but dose adjustment increments are lower for FCT than for DT (3.5–7 mg/kg for FCT; 5–10 mg/kg for DT), with maximum dose of 28 mg/kg/day for FCT. Transfusion-dependent patients should have SF monitored monthly and dose adjustments every 3 to 6 months based on SF trends, any weight change, therapeutic goals, and tolerability. Patients with NTDT should have SF monitored monthly and liver iron concentration monitored every 6 months (or if SF < 300 μg/L) with dose adjustment as needed. In renally impaired patients, the starting dose of FCT should be reduced by 50%. *Conclusions:* The algorithm should familiarize APs with new deferasirox formulations and educate them on appropriate administration and titration of these agents to enhance therapeutic compliance. This may lead to improved organ function and survival benefits among patients.

*Recommendations:* The algorithm should be published and distributed to APs to maximize its educational value among this audience.

## JL503. Chimeric Antigen Receptor T-Cell Therapy: Management of Toxicities

Harolynn K. Adeniran, FNP-C, Karla Ow, FNP-C, and Evelyn Acosta, FNP-C; The University of Texas MD Anderson Cancer Center, Houston, Texas

*Introduction:* Relapsed and/or refractory aggressive non-Hodgkin lymphoma can be devastating news to the lymphoma patient. In fact, outcomes for patients with refractory diffuse large B-cell lymphoma (DLBCL) are poor. The median overall survival with salvage chemotherapy is 6.6 months. Considerable effort has been given to the development of immunotherapy in this patient population. In particular, chimeric antigen receptor T (CAR-T) cell therapy targeting CD19 is being evaluated for various disease states including non-Hodgkin lymphoma. Phase 1 and 2 trials have demonstrated that CD19-specific CAR-T cell therapy is emerging as a potential new tool in the management of B-cell malignancies. CAR-T cells are generated by genetically programming autologous T cells to express CAR molecules that target CD19 antigen on tumor cells. While it is highly efficacious, CAR-T cell therapy can be associated with potentially life threatening toxicities. These toxicities result from T cell activation leading to a profound inflammatory response and cytokine release. Cytokine release syndrome (CRS) most commonly manifests as fever, hypotension, neurotoxicity, coagulopathy, and multi-organ dysfunction.

*Description:* 67-year-old male diagnosed with stage IIE DLBCL in 2003 with mesenteric and small bowel disease. He later relapsed and underwent autologous stem cell transplant with R-BEAM in 2/2014. In 12/2014 he again relapsed with mesenteric lymph node involvement and was later enrolled on CD19 CAR-T cell study. Following conditioning therapy with cyclophosphamide and fludarabine, he received CAR-T cells by intravenous infusion. His course was complicated by CRS symptoms including fever, hypotension, and altered mental status. He received supportive care for fevers with acetaminophen and cooling blanket. Antibiotics were given for neutropenia and urinary tract infection. Tocilizumab, an anti-IL-6 receptor antibody was given for grade 2 CRS. Fluid blouses were given for hypotension. He was also started on dexamethasone for neurotoxicity. He also received intrathecal hydrocortisone. His mentation returned to baseline on day 10. Patient achieved complete remission by PET-CT scan at 30 days after CAR-T infusion and remains in remission 2 years later.

*Conclusion:* Chemotherapy–refractory DLBCL is considered incurable and universally fatal, except for those who successfully undergo allogenic stem cell transplant. CAR-T cell therapy has shown promising treatment in refractory B-cell malignancies. This new therapy presents unique challenges in toxicity management. Advance nurse practitioners are at the forefront of this emerging therapy and must be familiar with the hallmark side effects and be prepared to manage them effectively in a timely manner to improve patient outcomes.

## JL504. Complex Illness Support Alongside Standard Oncology Care for Patients With Incurable Cancer

Kimberly A. Bland, DNP, APRN-NP, FNP, AOCN®, Nebraska Methodist Hospital, Omaha, Nebraska, and Alice Kindschuh, DNP, APRN-CNS, AGCNS-BC, CNE, Nebraska Methodist Health System and Nebraska Methodist College, Omaha, Nebraska

*Objective:* This was a descriptive study to evaluate the effect of Complex Illness Support (Palliative Care) alongside standard oncology care for patients with incurable cancer on symptom control, patient satisfaction with care, and chemotherapy utilization within 30 days of death using inferential statistics.

*Method:* A convenience sample of consecutive patients who presented through a lung cancer clinic in a Midwestern urban community hospital was utilized. One survey evaluated patient satisfaction with Complex Illness Support. Patients’ self-report of overall symptom burden (mild, moderate, severe) was documented at the first consultation visit and at the 3-month follow-up visit. For those patients who died within the study period, the electronic medical record was reviewed to determine chemotherapy utilization within 30 days of the patient’s death.

*Results:* A total of 13 physicians referred 22 patients with terminal illness to Complex Illness Support for a total of 22 patient visits over a 5-month timeframe. Of the 18 patients seen, 10 died (56%). Patients were highly symptomatic and a variety of interventions were used for support. Symptoms on the two patients seen consistently remained stable from initial consultation to the 3-month follow-up visit. Chemotherapy use within 30 days of death (two of the four patients received chemotherapy) was within national benchmark measures, and patients strongly endorsed satisfaction with the Complex Illness Support team.

*Conclusion:* Patients with cancer frequently experience significant symptom burden and psychosocial distress. Patients and providers are accepting of and asking for outpatient Complex Illness Support to address these issues. In this 5-month project, 56% of patients referred to this service died, affirming the rationale. For holistic care, Complex Illness Support needs to be available to patients where they most often access oncology care: in the cancer center. Integration of Complex Illness Support as part of standard oncologic care enhances patient care and satisfaction.

*Implications:* Complex Illness Support with an APRN provider alongside standard oncology care appears to be acceptable, beneficial, and feasible. Cancer patients can be co-managed between Complex Illness Support and oncology, with referral to other services as needed. Complex Illness Support must be offered where the patient receives care in order to be successful; oncology nurse practitioners are a critical element of this team.

## JL505. Coping Strategies of Hematology/Oncology Nurse Practitioners

Laura Bourdeanu, PhD, Excelsior College, Albany, New York, Shiyun Mai, MS, George Washington University, Washington, DC, and Arlene Pericak, MS, DA, FNP-C, FAANP, George Washington University, Washington, DC

*Background:* Providing care to hematology or oncology patients often requires dramatic shifts in attitudes and therapeutic interventions, as patients frequently transition from treatment to palliative care to hospice end-of-life care. NPs are routinely involved in implementing these changes in therapeutic goals, which requires a conscious move from the "cure" mode to that of recognizing death’s inevitability and providing comfort care. The purpose of this study is to explore the coping experiences of hematology/oncology NPs.

*Methods. Design:* Quantitative, descriptive cross sectional. *Sample:* Hematology/oncology nurse practitioners currently employed in the clinical setting. *Instruments:* Demographic characteristics, Gupsta’s Coping Skills Questionnaire. 

*Procedure:* NPs identified from Oncology Nursing Society (ONS) were contacted via email. The email included the introductory letter, the link to the survey, and a statement asking the nurse practitioners to forward the letter to their colleagues. 

*Results:* A total of 201 hematology/oncology NPs participated in the study; of these, 8 were eliminated from the analysis due to retirement or not currently working. The age of the participants ranged from 28 to 70 years (median 50 years). The sample was predominantly female (98%), married (72.5%), and participants had an MSN as a terminal degree (85%). The most common coping skills included "Maintain sense of control over work responsibilities" (99.5%), "Maintain sense of humor" (97.9%), "Maintain balance between professional and personal lives" (97.9%), and "Spend time with spouse/partner/family" (97.9%). Interestingly, 41.5% reported "Change practice specialty" as a way of coping with the job stressors. There was no significant difference in the coping skills in regard to age, education level, marital status, type of institution, and population served.

*Conclusions:* Hematology/oncology NPs use a variety of coping strategies, including maintaining a sense of control over work responsibilities, humor, maintaining balance between professional and personal lives to cope with patients’ transitions in treatment and/or care. Recommendations: Although hematology/oncology NPs are dynamic and resourceful when responding to challenging cancer patient care situations, collaboration between the NPs and support within their institution is essential in order to provide continuous coping assistance.

## JL506. Dedicated Advanced Practice Provider in an Outpatient Oncology Infusion Center

Christina Z. Page, MSN, RN, AOCNP®, AGPCNP-BC, and Delores P. McNair, MHA; Duke Cancer Institute, Durham, North Carolina

*Background:* Adverse drug reactions are a part of infusion treatment in oncology despite the best clinical efforts. At a community-based cancer center, an estimated 50 to 70 patients per day receive chemotherapy, immunotherapy, or non-oncology infusion. The incidence of chemotherapy-related hypersensitivity is about 5% (Gobel, 2005). The incidence of hypersensitivity reaction with immunotherapy can be higher than 15% with first dose of some monoclonal antibodies (Lenz, 2007). Adverse drug reactions (ADRs) have sizeable clinical costs (Sultana, Cutroneo, & Trifiro, 2013). A previous study by the Duke Raleigh Cancer Center demonstrated that the addition of an advanced practice provider (APP) in the outpatient infusion therapy center offered benefits of decreasing escalation of ADRs and the number of severe allergic reactions (Young, Dill, Fesko, & MacDiarmada, 2016). Leadership added a dedicated APP to the infusion room and a study was initiated to evaluate the impact and value added to the multidisciplinary groups who contribute to care of patients receiving outpatient infusions.

*Methods:* We initiated a 12-statement survey evaluated on a five-point Likert scale with two open-ended questions. Seventy-six members were surveyed within the cancer center to include infusion and clinic nurses, doctors, APPs, administrators, pharmacy and others. Data is being collected on the severity of ADRs.

*Results:* Survey response rate was 38%. Several survey questions showed 100% of respondents either agreed or strongly agreed to the following statements: the dedicated infusion APP decreases the level of stress and anxiety among patients and nursing staff during an acute infusion reaction, promotes patient safety, responds in a timely manner, manages ADRs appropriately, and is a positive presence in the infusion area. Most agreed that the APP minimized disruption of clinic flow. The survey encompassed a 5-month period in which approximately 45 infusion-related ADRs were identified with only 4 resulting in escalation of care to the Emergency Room.

*Conclusions:* Data demonstrates the positive impact and value of a dedicated infusion APP. Improvements in the management of ADRs offers benefits to the patient and likely cost savings to the institution. Limitations to this study include response rate, lack of comparative surveys, and limited data collection sources for ADRs. *Recommendations:* Additional data needed to measure the impact on patient safety, costs and sustainability of the role in the ever-changing arena of oncology treatment, as well as physician attitudes toward APP roles. Exploration of dedicated APPs in the infusion room by other institutions to evaluate the benefits.

## JL507. Development of a Professional Practice Model for Neuro-Oncology Advanced Practitioners at an Academic Medical Center

Christina Cone, DNP, APRN, ANP-BC, AOCNP®, and Mary Lou Affronti, DNP, APRN, ANP, MHSc; The Preston Robert Tisch Brain Tumor Center, Duke University Medical Center, Durham, North Carolina

**OUTSTANDING POSTER AWARD**

*Background:* In medicine, neuro-oncology practice falls outside the scope of established practice requirements for the specialties of neurology, medical oncology, and neurosurgery, justifying the prerequisite of specialized training to practice neuro-oncology. Neuro-oncology advanced practitioners (AP) also require specialization beyond the scope of population-based generalist training and education. There are no specialty standards or certifications for neuro-oncology APs, no formal training programs, and no literature that addresses the competency requirements of neuro-oncology APs. This quality improvement project’s primary purpose was to develop a professional practice model (PPM) for APs employed at an academic medical center (AMC) ambulatory neuro-oncology practice. Secondarily, the PPM would not only facilitate onboarding and training of new providers but also offer a standard against which to measure performance.

*Methods:* Using the Focus, Analyze, Develop, Execute, and Evaluate (FADE) quality improvement methodology the authors (1) reviewed literature and relevant professional organizations to identify possible professional competencies for neuro-oncology APs; (2) analyzed data to develop evidenced-based practice domains; (3) used purposive sampling to recruit an interprofessional team of neuro-oncology experts; (4) conducted a Delphi study with the interprofessional team of experts to gain consensus on practice domains and professional competencies; and finally (5) utilized the Delphi study results to create a PPM for neuro-oncology APs.

*Results:* Twenty-three participants (n = 23) were recruited for the Delphi study which was executed via electronic transmission using the Web-based software Qualtrics. Of the participants, 39% were physicians, 57% were APs, and 4% administrative. Seventy-eight percent of participants completed the Delphi round one and 52% completed the second round. After two rounds of the Delphi, the expert team reached consensus on six domains of practice with fifty corresponding competency statements. Domains of practice included medical knowledge, patient care, practice-based learning and improvement, interprofessional collaboration and communication skills, professionalism, and systems-based practice.

*Summary:* Through interprofessional collaboration and consensus, this quality improvement project successfully created a PPM for an AMC neuro-oncology AP team.

*Implications:* The PPM supports neuro-oncology APs by validating the unique set of skills that combines several specialties. The PPM provided the framework to standardize orientation and training, evaluate performance, and support the professional development of an AMC neuro-oncology AP team. The PPM may improve the patient experience through assurance of competent patient-centered care and may decrease per capita cost by enhancing retention of highly qualified staff, which in turn could save the health system thousands of dollars.

## JL508. Diversity Awareness and Documentation Practices Among Oncology Advanced Practice Providers: A Prospective Quality Improvement Project

Victoria Poillucci, MSN, ACNP-BC, and Christina Z. Page, MSN, RN, AOCNP®, AGPCNP-BC; Duke Cancer Institute, Durham, North Carolina

*Background:* Health-care providers have the challenge of caring for widely diverse populations. The United States currently has the most polyracial, polyethnic, and polyreligious population in history (Andrews & Boyle, 2002). The 2002 Institute of Medicine (IOM) landmark report titled Unequal Treatment: Confronting Racial and Ethnic Disparities in Health Care confirmed that disparities in health care exist and are associated with worse outcomes. Findings also suggest that bias, prejudice and stereotyping by the health care provider may contribute to disparities. The IOM called for efforts to address these disparities, including increasing awareness among health care providers (Institute of Medicine [IOM], 2003).

*Objective:* To assess the cultural self-awareness of oncology advanced practice providers (APPs) who practice in a community- based outpatient cancer center. To investigate the extent to which oncology APPs include cultural care into patient assessments.

*Methods:* APPs working in an academic community-based hospital outpatient oncology clinic completed a questionnaire titled, the "Looking Glass Examination." This tool evaluates cultural self-awareness and examines the intrinsic attitudes that providers may have when caring for patients of diverse backgrounds (Yeo et al, 2011). A prospective, quality improvement chart review was performed to analyze the extent to which cultural themes were addressed during oncology clinic visits. A list of cultural keywords and phrases was used as a guide for chart review. Ten percent, 164 patients, of the 2015 cancer population at the institution were examined, which included a stratified sample of the top five disease groups: breast, lung, gastrointestinal, genitourinary, hematologic, and gynecologic cancers (Duke Cancer Institute, 2016). 

*Results:* Responses from the questionnaire were analyzed. There was a 92% response rate. Forty-five percent of APPs demonstrate above average cultural awareness. Upon chart review, of the 28 cultural keyword items, an average of 4.88 items were addressed each visit, including age and gender. Multiple cultural items, including literacy, language, use of herbal treatments, insurance status, and belief about disease were addressed less than 5% of the time.

*Conclusions:* Oncology APPs show high cultural self-awareness. We predict the cultural documentation will be low. Limitations to this study include potential bias of the APP after completing the questionnaire and inter-reviewer variability. There may be cultural aspects addressed during the clinic visit that were not documented.

*Recommendations:* Cultural assessment should be a standard part of oncology patient assessments. An open-ended cultural questionnaire would help APPs better assess the cultural needs of their patients. A cultural review of systems dot phrase was created for use in the EMR. This can be shared and applied across the health system to better serve our patients. 

## JL509. Empowering the Advanced Practitioner to Champion Education of Tumor Lysis Syndrome to the Clinical Nursing Team

Allister B. Chase, MSN, RN, FNP-BC, AOCNP®, Karen Groth, MSN, RN, CNS, ARNP, Colleen Weber, BSN, RN, Linda Schnell, MSN, RN, OCN®, and Loretta Morehead, BSN, RN; Genentech Hematology

*Context:* A metabolic emergency related to malignancy, tumor lysis syndrome (TLS) signals a potentially life-threatening process in which prevention and surveillance are key to positive patient outcomes. Laboratory disturbances that characterize the onset of TLS can include one or more of the following metabolic triggers: hyperkalemia, hyperuricemia, hypocalcemia, and hyperphosphatemia. Heralding a rapid turnover of cellular breakdown, such metabolic disturbances can occur both spontaneously and following treatment initiation, especially with aggressive and/or hematologic malignancies.

*Approach:* Within the current treatment landscape, targeted therapies are diversifying options for cancer patients. With the development of new targeted oncology drugs TLS has also been observed in patients with cancers that were previously rarely associated with this complication. TLS is a potential complication with patients receiving intravenous therapies, as well as oral agents. As each new therapy brings its own set of management concerns, it is paramount that advanced practitioners (APs) and nurses stay abreast of each regimen’s unique mechanism of action and potential side effects, including TLS. Oncology APs play a critical role in the patient management and prevention of TLS, though it is essential that the entire nursing team be prepared to adequately assess and prevent this life-threatening consequence. *Aim:* Empower the AP to champion the awareness, education, and mitigation strategies among APs and nursing staff in order to reduce the occurrence and/or effects of TLS.

*Summary:* Effective management and prevention of TLS necessitates that APs take ownership in the development of an educational plan for their nursing colleagues. An initial survey of the nursing staff may assess their current foundation and understanding of this metabolic disorder. This assessment would include their perception of the pathophysiology, nursing implications, and the individual patient management and education strategies. Building upon this foundation, the AP will work with team educators to develop a learning plan that will accommodate for the current and future treatment landscape enabling the nursing staff to assimilate this knowledge into their patient care. This poster will highlight the educational content that is essential for TLS risk assessment and preventative measures.

*Implications:* Within their unique role and experience, APs must take the lead in raising awareness of emerging treatments and how they may impact the patient’s risk for TLS. APs can influence patient outcomes through the management of treatment plans, education of patients, AND influencing their nursing colleagues’ knowledge of risk assessment and patient care needs.

## JL510. Febrile Neutropenia Risk Assessment Prior to Chemotherapy and Use of Colony-Stimulating Factor Prophylaxis in U.S. Community Oncology Clinics

Soeren Mattke, MD, MPH, DSc, RAND Health Advisory Services, RAND Corporation, Boston, Massachusetts, Elizabeth M. Sloss, PhD, RAND Corporation, Arlington, Virginia, Nabeel S. Qureshi, MPH, RAND Corporation, Boston, Massachusetts, Carol Roth, RN, MPH, RAND Corporation, Santa Monica, California, and Jonathan W. W. Goldman, MD, University of California Los Angeles, Los Angeles, California

*Objective:* Current guidelines recommend use of prophylactic colony-stimulating factor (CSF) prior to chemotherapy to prevent febrile neutropenia (FN) if the FN risk is greater than 20%. Use of CSF in lower-risk patients has been identified as low-value care in ASCO’s "Choosing Wisely" recommendations. Implementing those recommendations requires FN risk assessment because FN risk depends on the interplay of patient-level risk factors and the inherent toxicity of the regimen. We investigated the rate at which such risk assessment is documented outside of clinical trials and the implications for CSF use decisions.

*Methods:* We conducted retrospective chart review of 192 patients in five community oncology clinics in the U.S. prior to the first chemotherapy cycle to ascertain whether FN risk assessment was documented. We determined regimen risk based on published literature. Any documented reference to FN risk was categorized as having performed the risk assessment. *Results:* FN risk assessment was documented in 13% of patients across the five clinics with a range from 0% to 27%. The probability of FN risk assessment did not vary significantly by age, sex, race/ethnicity, and type of insurance coverage, but was significantly higher for chemotherapy regimens with an inherent risk of greater than 20% (21% vs. 7%, *p* < .01). 54% of patients (103/192) received CSF prophylaxis and patients were twice as likely to receive CSF if risk assessment was documented (96% vs. 48%, *p* < .01), even after accounting for regimen risk. 43% of patients (53/122) with low-risk regimens received CSF prophylaxis and 29% of patients (20/70) with high-risk regimens did not.

*Conclusions:* FN risk assessment to inform appropriate use of CSF prior to initiation of chemotherapy was infrequently documented in our sample of records from U.S. community oncology clinics. Failure to document FN risk may have contributed to both overuse and underuse of CSF prophylaxis.

*Recommendations:* Increased use of FN risk assessment prior to initiation of chemotherapy could help to improve safety of chemotherapy and avoid low-value utilization.

## JL511. Hematology/Oncology Nurse Practitioners: A Study of Job Satisfaction, Stress, and Intention to Leave the Specialty

Laura Bourdeanu, PhD, Excelsior College, Albany, New York, Shiyun Mai, MS, George Washington University, Washington, DC, Yuan Shen, MS, George Washington University, Washington, DC, Haoqi Sun, MS, George Washington University, Washington, DC, Suya Wang, MS, George Washington University, Washington, DC, and Karen Morrissey, MSN, CVS

*Background:* The predicted oncology/hematology workforce shortages are likely to place greater demands upon nurse practitioners (NP), as the proposed solution to this shortage is expanding the role of the nurse practitioner. This may lead to an increase in work-related stress and decrease in job satisfaction. The purpose of this study is to determine the hematology/oncology NP’s job satisfaction/stress, and their intent to leave their job or profession in order to better decide the further development and expansion of the NP role.

*Methods. Design:* Quantitative descriptive cross sectional. *Sample:* Hematology/oncology NPs currently employed in the clinical setting. *Instruments:* Demographic characteristics, the Intent to Leave Scale, and the Hospital Consultants’ Job Stress & Satisfaction Questionnaire.

*Procedure:* Emails including the request for participation letter, link to the survey, and a statement asking the nurse practitioners to forward the letter to their colleagues were sent to eligible participants obtained from Oncology Nursing Society. *Results:* A total of 193 eligible hematology/oncology NPs participated in the study. The age of the participants ranged from 28 to 70 years (median 50 years). The sample was predominantly female (98%), married (72.5%), and had an MSN as a terminal degree (85%). Overall, 79.3% of the NPs reported being quite a bit or a lot satisfied with their job; predominantly due to having a high level of responsibility (81.4%) and a good relationship with the patients (93.3%). Of the 13.5% NPs who reported quite a bit or a lot of stress, 45.6% claimed high sources of stress were related to being involved with the emotional distress of patients and 40.4% related to disruption of home life through spending long hours at work. Of the all participants, 14% were actively searching for an alternative to the profession, 10.4% will look for a new job outside the profession in the near future and 11.4% will leave the profession when they can. High stress, low job satisfaction, and inability to schedule days off were predictors of the NPs’ intent to leave the profession.

*Conclusions:* The results of this study found that nearly 15% of the hematology/oncology NPs intend to leave the profession. Job satisfaction/stress and inability to schedule days off are significant predictors for intent to leave in this profession.

*Recommendations:* These findings can be utilized by organizations to enhance job satisfaction, reduce stress, help to secure days off as needed, and use these as predictors for intent to leave.

## JL512. Impact Analysis of an Advanced Practice Nurse on Outcomes for an Inpatient Hematological Malignancies Service in an Academic Medical Center

Sarah J. Jimenez, MSN, APN-C, AOCNP®, Rutgers Cancer Institute of New Jersey, New Brunswick, New Jersey, Tracy Krimmel, DNP, APN-BC, AOCN®, Rutgers Cancer Institute of New Jersey, New Brunswick, New Jersey, and Kimyatta Washington, MHSA, Department of Oncology Services, RWJBarnabas Health, West Orange, New Jersey

*Background:* The complex care and management of hematological malignancy patients in the acute care setting requires a multidisciplinary team approach whose aim is to ensure favorable outcomes and safe discharge for the patients. Historically, the care team at our institution consisted of one attending physician, one oncology fellow, and one to two medical residents, all of which rotated frequently through the service. An analysis of the program showed that prolonged length of stay and overall quality of care were noted to be areas of improvement. Factors that contributed to prolonged length of stay and level of quality care included: no defined process of admission/discharge, lack of consistent team members, lack of consistency in care between providers, lack of communication between the inpatient/outpatient teams, lack of social work/case management support, disparities in documentation, and coding errors. As a quality assurance initiative, employment of an advanced practice nurse (APN) was suggested to help improve overall quality of care.

*Intervention:* An oncology APN was employed to oversee the management of patients admitted to the hematological malignancies service. The APN was the only consistent member of the service. The APN facilitated the admissions/discharges for all patients on the service, acted as liaison between inpatient and outpatient teams, developed standardized order sets, implemented multidisciplinary rounds to include both inpatient and outpatient social workers, case managers, nurse navigator, and oncology pharmacist. Thorough analysis of documentation led to the development of standardized progress note templates to ensure adequate documentation and billing capture. The coding team was re-educated to understand proper coding and a primary coder was provided to the team.

*Outcomes:* Retrospective data analysis over a 2-year period (2015–2016) revealed the implementation of an APN resulted in decreased length of stay, decreased cost of hospitalization per patient/per stay, better communication between inpatient/outpatient teams, and increased provider satisfaction. Specific data will be included in final poster.

*Conclusion/Implications to Practice:* The use of APNs for complex patients such as those with hematological malignancies have been shown to have improved outcomes, lower cost of hospitalization, and shorter lengths of stay when compared to historical data. In the setting of limited resources, APNs play a pivotal role in providing affordable quality complex patient care. More studies are needed to evaluate the role of advanced practice providers (APP) in the hematological malignancies inpatient service. Expansion of the APP specialized service and employment of additional APPs may continue to improve both patient and provider experience.

## JL513. Improving Data Quality and Knowledge Management Through the Application of Artificial Intelligence to Oncology Care Model Patients

Jacqueline T. Norrell, DNP, BS-CIS, FNP-BC, Renee Kurz, DNP, MSN, FNP-BC, Janet Gordils-Perez, DNP, ANP-BC, AOCNP®, and Doris Fonseca, BS Public Health; Rutgers Cancer Institute of New Jersey, New Brunswick, New Jersey

*Background:* The Center for Medicare Services Oncology Care Model (OCM) is defined as a quality improvement initiative focused on improving care for Medicare patients receiving chemotherapy. The six CMS requirements include: provide patient navigation; document a care plan containing the 13 components in the IOM Care Management Plan; provide 24/7 symptom management access; treat patients with therapies consistent with national guidelines; use data to drive continuous quality improvement; and use an ONC-certified EHR. Rutgers Cancer Institute as the only NCI-designated CCC in NJ, a state that ranks among the worst for overall cancer incidence, is a critical resource for NJ. In keeping with its mission, the Institute seeks to continually provide new clinical programs and participate in value-based payment models to meet the needs of cancer patients to improve quality care in a cost-efficient manner. In May of 2015, the Cancer Institute pursued participation in the OCM by submitting an application and in April of 2016, was chosen to participate.

*Methods:* An executive core steering committee was formed to strategize operational processes to meet all OCM requirements. The subcommittee, Quality Reporting Committee’s (QRC), overarching initial goal was to develop the tracking mechanism to assess the quality of submitted OCM data for validity, accuracy, and completeness, and make recommendations for remediation of data quality issues to facilitate reporting of OCM measures. The methodology included an evaluation of all clinical systems, workflow, and complex structured data. Statistical analysis was performed, a gap analysis completed, reports created, and a scorecard developed that identified data with steps for remedial action. The QRC and OCM team members rectified the data quality issues.

*Results:* Results revealed limitations of the legacy EMR including missing, undefined, or inaccessible data elements, precluding OCM reporting or analysis. By correcting data quality issues, there was an improvement in the quality of care patients received by the organization and the Advanced Practice Nurse (APN).

*Conclusions:* Data democratization, robotic process automation (RPM), and the use of artificial intelligence (AI) facilitated our ability to predict, rather than react, to newly discovered data quality issues. Guided by the OCM framework, the use of RPM and AI provided an efficient method of data capture and analysis for reporting OCM measures.

*Recommendations:* Ongoing QA practices of OCM measures using AI, and RPM, improving data governance and clinical informatics, and implementing an information-based strategy to address the quality of oncology service delivery by the organization is essential.

## JL515. Intra-Professional Collaboration for Safe Chemotherapy Administration

Stephanie L. Jackson, MSN, RN, AOCNS®, BMTCN, Ronald Reagan UCLA Medical Center, Los Angeles, California

*Background:* With continuous advancements in science, technology, and medicine, surgical patients are offered increasingly complex treatment options including intraoperative administration of chemotherapy. The expanded use of cytotoxic medications across multiple surgical specialty services in the operating room lead to a need to standardize practices related to chemotherapy administration and safe handling practice. A collaborative group of advance practice nurses created an evidenced-based standard of practice to ensure optimal patient outcomes and a safe practice environment.

*Methods:* Based on a review of the literature, the Oncology Nursing Society (ONS) standards and National Institute of Occupational Safety and Health (NIOSH) regulations, a comprehensive evidence-based standard of practice for safe intraoperative chemotherapy administration and handling was developed. The clinical improvements specifically focused on in-suite preparation and administration of chemotherapy, correct use of approved chemotherapy-rated personal protective equipment, exposure and spill management, and contaminated waste and instrument handling. In addition, processes were developed for documentation of intraoperative verification of these high-risk medications, as well as formalized communication of chemotherapy precautions during the handover transfer report. Because different levels of knowledge produce variations in practice, educational in-services were provided to the perioperative nurses and surgical technologists. To further support the clinical staff’s understanding of the bundled practice change, this information was reinforced by developing a competency assessment tool; incorporating the content into skills day; revising the electronically accessible policy and procedure; protocol development; and highlighting critical elements in the service-specific doctors’ preference cards.

*Conclusions:* An evaluation based on the Likert scale was given to the nurses as well as surgical technicians after their skills lab. Ninety percent of the staff felt the competency training was very valuable and 10% felt it was valuable. Quarterly needs assessments are provided to the staff to sustain their knowledge and hands-on competency.

*Recommendations:* The perioperative nursing practice implications have been knowledge enhancement, increased confidence in professional practice, and promotion of value through reducing redundancy in efforts and workflow. Through re-evaluation of the standard of practice and clinical staff feedback, we were able to evaluate compliance, sustainability, and furthermore, identify opportunities for additional innovative processes related to chemotherapy safety in the operating room. As the field of oncology continues to grow and expand, it is inevitable that non-oncology nurses acquire the skill set to assess, monitor, and evaluate the oncology care provided to patients under their clinical care.

## JL516. Management of Cytokine Release Syndrome and Neurologic Events in ZUMA-1: Anti-CD19 CAR T-Cell Therapy, Axicabtagene Ciloleucel (axi-cel), in Patients With Refractory, Aggressive Non-Hodgkin Lymphoma

Sherry Adkins, RN, MSN, ANP-C, The University of Texas MD Anderson Cancer Center, Houston, Texas, Amy Patterson, MSN, RN, AOCNS®, BMTCN, Moffitt Cancer Center, Tampa, Florida, Nicole Kahle, MS, RN, OCN®, BMTCN, Moffitt Cancer Center, Tampa, Florida, and Carol Sheridan, RN, MSN, OCN®, BMTCN, Montefiore Medical Center, Bronx, New York

*Background:* Chimeric antigen receptor (CAR) T-cell therapy is a promising treatment option for patients with refractory aggressive non-Hodgkin lymphoma (NHL). ZUMA-1, a pivotal, multicenter trial of axi-cel resulted in an objective response rate of 82% (54% complete responses; Locke et al., 2017). CAR T-cell therapy is associated with cytokine release syndrome (CRS) and neurologic events (NE). Advanced practitioners (APs) should be familiar with the clinical presentation and management of CAR T cell-associated AEs.

*Methods:* Eligible patients were leukapheresed and received low-dose conditioning chemotherapy followed by axi-cel administration as inpatients. Patients were monitored for at least 7 days post-infusion in the hospital. APs at sites reviewed cases to compare multicenter approaches and collate experiences into informative, best practices.

*Results:* Ninety-three percent of patients experienced CRS (13% grade ≥ 3) and 64% experienced NE (28% grade ≥ 3). Nearly all CRS and NE was reversible. Thorough and consistent physical, laboratory, and neurologic assessments from baseline to discharge allowed for early identification of symptoms. For CRS, pyrexia was a common early symptom (76%), followed by hypotension (41%), hypoxia (22%), and tachycardia (21%). Familiarity with standardized CRS grading by unit nurses enabled rapid escalation to APs in cases of CRS progression. Current axi-cel CRS management guidance (Lee et al., 2014) recommends continuous cardiac telemetry and pulse oximetry for patients with grade 2 CRS; and grade ≥ 3 CRS requires management in a monitored care or intensive care unit. Patients requiring vasopressors (13%) for hypotension required telemetry monitoring. If available, telemetry was monitored within the treatment unit to reduce transfer to the intensive care unit for telemetry alone. Daily monitoring for elevated C-reactive protein and ferritin levels may identify patients at greater risk for severe CRS and hemophagocytic lymphohistiocytosis. NE onset was generally later than that of CRS. For NE, handwriting impairment often preceded common symptoms of encephalopathy (34%), confusion (29%), and tremor (29%). Case reports will be presented illustrating real-world experiences managing patients treated with CAR T-cell therapy.

*Summary:* Axi-cel has demonstrated significant clinical benefit but with a distinct toxicity profile associated with this class of therapy. While toxicities could be severe, they were generally manageable and reversible.

*Implications:* Close patient monitoring by unit nurses and early AE interventions by APs was critical to optimizing safety. Collaboration between departments and across sites allows continued refinement of management strategies, further optimizing care and improving the benefit:risk ratio of CAR T-cell therapy.

## JL517. Minimal Residual Disease in Hematological Malignancies: Implications for Practice

Mickey Stiger Register, FNP-BC, MSN, MPH, Genentech, Jennifer Giarratana, MSN, ANP, AOCNP®, Genentech, and Colleen Weber, BSN, RN, Genentech

*Background:* As advanced practice providers (APP), it is essential to determine the optimal management of a patient with a hematological malignancy. The efficacy of treatment options are evaluated in clinical trials using common endpoints, such as, overall survival, progression-free survival, and response rate. A review of more recent clinical trials and publications of hematological malignancies reveals there is an emerging focus on minimal residual disease (MRD) negativity as a secondary endpoint. MRD describes the lowest presence of malignant cells that are detectable using available methods. After completing treatment, a patient is deemed MRD negative when there is an absence of malignant cells by analysis using current sensitive testing modalities. In several hematologic cancers, MRD negativity is considered the single most predictor of overall survival and is therefore an important component of patient management.

*Intervention:* It is important for APPs to be aware of MRD as an endpoint in clinical trials for some hematologic malignancies. Understanding of the methods of measuring MRD will assist in comprehending the analysis of trial data. This information may assist in guiding treatment decisions and patient management, ultimately influencing practice implications.

*Findings:* MRD analysis requires testing of blood, bone marrow, and/or lymph nodes. The methodologies for MRD evaluation, including morphology, cell culture assays, karyotoypic analysis, fluorescence in situ hybridization (FISH) techniques; flow cytometry and immunophenotypic analyses; and molecular analyses (Southern blotting and PCR), will provide valuable clinical knowledge.

*Summary:* Understanding MRD methodologies and data analysis in clinical trials will assist the APP in managing patients with hematologic malignancies. This also provides the foundation for a thoughtful patient discussion regarding the prognosis and treatment options. In clinical practice, the presence or absence of residual disease may be a useful measurement in determining a patient’s response to treatment.

*Implications:* In some hematologic malignancies the presence of MRD after the completion of treatment has been shown to be associated with early relapse. Understanding this may be helpful in establishing the patient’s molecular response to therapy and options for future treatment. At this time, the majority of MRD testing is performed in clinical trials and further study for clinical application to general oncology practice is suggested in the literature. Yet, there is an increasing amount of discussion of MRD status in patient education literature and APPs should familiarize themselves with MRD as part of both disease management, patient monitoring, and discussion.

## JL518. Mistaken Identity: The Soft Tissue Sarcoma Conundrum

Allison Lock, MPAS, PA-C, The University of Texas MD Anderson Cancer Center, Houston, Texas

*Introduction:* Soft tissue sarcoma (STS) is a cancer of connective tissues and can occur anywhere in the body. Approximately 13,000 new cases are diagnosed in the US each year and about 5,000 annual deaths are attributed to this disease. With over 70 subtypes comprising about 1% of adult cancers, STS is considered rare. Coupled with its rarity and propensity to mimic benign etiologies, accurate diagnosis can be challenging and mismanagement is common. STS prognosis is largely contingent upon its initial management. NCCN Guidelines advise a multidisciplinary approach to management for improved clinical outcomes.

*Purpose:* (1) Highlight cases of STS with initial suspicion of benign etiologies; (2) Discuss the importance of considering STS in the differential diagnosis when evaluating a soft tissue mass; (3) Review NCCN Guidelines for STS management.

*Description:* A 60-year-old female presents to her gynecologist for evaluation of a left labial "knot." It is initially diagnosed as a sebaceous cyst. A 46-year-old female presents to her PCP for evaluation of a self-palpated painless, rubbery abdominal wall mass. Initial diagnosis is lipoma. A 58-year-old male presents to a general surgeon due to multiple recurrent fat containing inguinal hernias. This case series is presented to discuss commonly encountered scenarios of mistaken identities in soft tissue sarcoma. Each case will be discussed from initial presentation and diagnosis through eventual referral to a tertiary care center for diagnostic confirmation and management. Next, data is presented to support the idea that initial treatment of soft tissue sarcomas is critical to prognosis. Further, NCCN Guideline–derived strategies for more accurate primary diagnosis and management will be suggested.

*Discussion:* From sebaceous cysts, to lipomas, and hernias, STS is frequently mistaken for benign etiologies on initial presentation. Each case exemplifies a common clinical scenario that may be encountered in any specialty. It is important for APPs to recognize STS as a potential item on the differential diagnosis, as mismanagement may result in poor clinical outcomes.

## JL519. Modified Outpatient Regimen of Daratumumab to Reduce First Infusion Reaction Risk

Christopher Campen, PharmD, BCOP, Elizabeth H. Cull, MD, Brittany Wills, PharmD, Lise Langston, PharmD, Saeeda Chowdhury, MD, Susan Funk, NP, and Suzanne Fanning, DO; Greenville Health System Cancer Institute

*Background:* Daratumumab (dara) is a CD38 targeting monoclonal antibody with significant activity in multiple myeloma. Infusion-related reactions (IRRs) include cough, dyspnea, bronchospasm, and chills. Between 45% and 71% of patients experienced an IRR on study, mainly with the first treatment. The first treatment of daratumumab must be infused over a prolonged time period, which is difficult to accommodate in the outpatient setting. There is significant interest in reducing the risk of IRRs with the first treatment and providing a treatment schema that allows for outpatient administration in an 8-hour infusion schedule.

*Methods:* Standard IRR prophylaxis recommended by the manufacturer includes acetaminophen, a steroid, and an antihistamine prior to the infusion. In April 2016 our daratumumab protocol was modified to include additional IRR prophylaxis. Medications added to the standard included dexamethasone the night prior to first treatment with montelukast and famotidine the day of treatment. In order to facilitate outpatient administration, the first dose was split into two days (C1D1/D2) at a dose of 8 mg/kg/day (total 16 mg/kg).

*Results:* Nineteen patients received daratumumab at the institution, with 15 patients receiving the modified outpatient regimen from November 2015 to January 2017. No patients reported respiratory symptoms during C1D1/D2. The IRR frequency during C1D1/2 was 1/15 (7%). This single patient reported gastrointestinal symptoms and chills during the first infusion and was able to safely continue treatment.

*Conclusions:* Additional prophylaxis for IRRs and a modified split dose regimen may reduce the risk of IRR with dara and provide for safe outpatient administration with an 8-hour clinic schedule. In our experience, IRRs were significantly reduced when compared to the FDA registry trials. Knowledge of improved tolerance to therapy and easier administration may increase use of daratumumab in the community.

## JL520. Oncology Care Model and the Transformation of the APN Role in the Care of Patients Undergoing Chemotherapy

Elsie Castrorao, MSN, APN-C, OCN®, Renee Kurz, DNP, MSN, FNP-BC, Andrew D. Kass, MSN, AGNP-C, AOCNP®, Jacqueline T. Norrell, DNP, BS-CIS, FNP-BC, and Janet Gordils-Perez, DNP, ANP-BC, AOCNP®; Rutgers Cancer Institute of New Jersey, New Brunswick, New Jersey

*Objective/Background:* The Center for Medicare Services Oncology Care Model (OCM) is defined as a quality improvement initiative focused on improving care for Medicare patients receiving chemotherapy. The Rutgers Cancer Institute of New Jersey began participating in the program in July 2016. The institute’s primary objective was to align our practice to the OCM framework. A secondary objective was to employ the advance practice nurses (APNs) to enhance care coordination, ensure appropriateness of care, and provide quality comprehensive care, while at the same time reducing expenditures.

*Methods:* Resources were realigned to include two full-time APN positions whose function was to address acute symptom management. Treatment area hours were extended from 7am to 7pm. Service calls were directed to the APNs from 5pm to 7pm. The OCM APNs were relocated to an office adjacent to the treatment center to streamline communications and workflow. All staff members and physicians were educated on APN availability and services of OCM APNs. The OCM framework was incorporated into APN practice including a comprehensive symptom assessment and psychosocial evaluation. Questionnaires were developed to document symptoms, and meet the performance metrics for OCM. The assessment also included advanced care planning, depression and emotional distress surveys. Through the evaluation of clinical pathways, referral triggers were defined, and incorporated into the flow of clinical care.

*Results:* In a 13-month period, 500 patients were assessed. Approximately 200 patients with symptom management issues were advised with appropriate interventions. Eighty-eight patients were referred to our social work department for further evaluation of depression and suicidal ideation. Additionally, a downward trend of emergency room visits and hospital readmissions has been demonstrated since the inception of the OCM program.

*Conclusion:* Through the addition of the OCM APNs, the institution’s practice was successfully transformed and aligned to meet the OCM guidelines. Utilization of the OCM questionnaires provided a structured approach to the delivery of comprehensive care. The OCM APNs were able to address, remediate, and share the responsibility of psychosocial issues, and improve the quality of care of our patients. Symptom management response time has been reduced.

*Recommendations:* It would be beneficial clinically and financially to expand the OCM model to all of our patients throughout the institution. In addition, the creation of a dedicated urgent care clinic for patients to access on non-treatment days would yield multiple benefits.

## JL521. Oncology Nurse Practitioners’ Beliefs About Evidence-Based Practice and Barriers to Research Utilization

Rita M. Jakubowski, DNP, ANP-BC, BMTCN, Mount Sinai Hospital, New York, New York

*Purpose/Objectives:* To determine the relationship between nurse practitioners’ (NP) beliefs about evidence-based practice (EBP) and perceived barriers to research utilization within their practice. Four categories of barriers: characteristics of the adopter, organizational structure, quality of research and communication, and accessibility of research, as it relates to NP beliefs about research utilization were explored.

*Design:* Demographic and validated surveys for EBP and research utilization were converted to web-based format using Survey Monkey. Results are presented descriptively.

*Setting:* 1,171-bed tertiary and quarternary care Magnet-designated urban, teaching hospital with NCI cancer center designation and FACT accredited stem cell transplant program.

*Sample:* A convenience sample of 27 NPs was recruited from inpatient and outpatient practices (Hematology, Medical Oncology, and Bone Marrow Transplant) during February 2017.

*Methods:* A correlational quantitative survey including area of practice, professional background, years in practice, certification, previous experience in EBP, and knowledge of a practice mentor were summarized using descriptive statistics. Surveys utilized: Funk, Champagne, Weise, and Tornquist’s Barriers to Research Utilization Survey and Melnyk, Fineout-Overholt, and Mays’ EBP Belief Survey.

*Findings:* NPs value EBP; however, barriers exist to its implementation. Although EBP and research utilization may have been a focus in their education, only ~1/3 reported participation in an EBP project within 1 year. There was no statistically significant relationship between NP beliefs about EBP and barriers to research utilization as reflected in 4 categories: adopter, organization, research, accessibility and communication. However, primary barriers to research utilization were identified. While > 50% reported feeling confident in implementing EBP, are clear about the steps in EBP and ability to implement it in a time-efficient way, only ~25% reported they knew how to implement EBP sufficiently to change practice. The top five barriers to research utilization included: "the nurse..." "...does not feel she/he has the authority to change patient care procedures," "... does not have time to read research," "...does not feel capable of evaluating the quality of the research;" "there is insufficient time on the job to implement new ideas," "the relevant literature is not compiled in one place."

*Conclusions:* NPs value EBP; however, dedicated time for NPs to participate in EBP projects needs to be acknowledged and supported, and NPs need to feel empowered by the organization to utilize research and EBP to change clinical practice. Mentorship may assist in efficient collection and interpretation of research results and in guiding the integration of these results into their practice.

## JL522. Optimizing Survivorship Care Plan Quality and Efficiency Through Cancer Registry Partnerships

Karen J. Hammelef, RN, DNP, Carevive Systems, Inc, Carrie Stricker, PhD, RN, Carevive Systems, Inc, Carrie Tilley, RN, MS, ANP, AOCNP®, Carevive Systems, Inc, Margaret A. O’Grady, RN, MSN, OCN®, Abington Memorial Hospital, Abington, Pennsylvania, Noel M. Arring, DNP, RN, OCN®, Mayo Clinic, Phoenix, Arizona, and Deborah A. Walker, RN, MSN, APRN®, Hartford Health Center, Hartford, Connecticut

*Objective:* Providing survivorship care plans (SCPs)to cancer patients was initially endorsed by the Institute of Medicine a decade ago as an essential component of quality care. Now mandated by accreditation and value-based payment programs, the process of care plan development and provision remains fraught with barriers, including mining of data necessary to generate a patient-specific treatment summary (TS) and care plan, and a mechanism for tailoring SCP content to individual clinical contexts. Electronic medical records (EMRs) facilitate the process but provide numerous limitations including lack of standardized data fields, inadequate tracking and reporting mechanisms, and sparse and disparate content, given the tremendous time and expertise needed to develop and maintain cancer-specific content. SCP software that is integrated with registry has shown promise to efficiently deliver a high volume of quality SCPs and allow clinicians to focus on patient care rather than manual creation.

*Methods:* Four diverse community and academic US cancer centers implemented SCP delivery utilizing cancer registry as the data source, interfaced by a commercially available rules-driven platform that ensures individualized, evidence-based care plan content for each patient. While all share this common platform, their processes for leveraging these capabilities and integrating the registry team into workflow vary widely and will be qualitatively described. Numbers of SCPs delivered and time spent in preparation will be described and compared pre and post registry-involvement with SCPs.

*Results:* Three of the 4 institutions have completed implementation and have been actively providing SCPs for between 3 and 24 months. Total SCP delivery/year reached 621 in one site, quadrupling rates from prior. Mean number of SCPs per month across established centers currently is 38 (range 21–101), time preparing SCPs was reduced to 5 to 20 minutes post registry-integrated processes from up to 2 hours at baseline. Variable workflow processes and roles for integrating registry staff will be described in the presentation.

*Conclusions:* Cancer registry data paired with an evidenced-based rules driven survivorship care planning software is an efficient solution to delivery of tailored and evidence based SCPs. Each of 4 centers have identified barriers and facilitators to this process that include degree and timing of registrar involvement, identified registrar champions and prior experience with cancer registry data. These and other barriers and facilitators to the process offer important implications for the advanced practice nurse seeking to implement this process.

## JL523. Outcome Evaluation of Timing Recommendations for Influenza Vaccinations in Patients Receiving Intravenous Chemotherapy

Elizabeth A. Hageman, PharmD, BCPS, Cayuga Medical Center, Ithaca, New York, Alex D. Vondracek, PharmD, Cayuga Medical Center, Ithaca, New York, and Ryan Mack, Albany College of Pharmacy and Health Sciences, Albany, New York

*Objective:* The Centers for Disease Control and Prevention (CDC) and the Infectious Disease Society of America (IDSA) recommend vaccinating cancer patients against influenza 2 weeks prior to the initiation of chemotherapy in order for a patient to acquire seroprotection. However, this recommendation does not address optimal timing of vaccine administration for patients already receiving intravenous (IV) chemotherapy during the flu season. Cayuga Hematology Oncology Associates (CHOA) created vaccine timing recommendations with the goal of administration in the middle of chemotherapy cycles to avoid the need for immunologic response during a patient’s nadir. These recommendations were put into practice during the 2016 to 2017 flu season. The purpose of this evaluation was to determine if the timing of the vaccine in the middle of the chemotherapy cycle resulted in fewer confirmed influenza infections.

*Methods:* A retrospective review of patients from the 2014 to 2015 and 2016 to 2017 influenza season compared the timing of the vaccination within 14-, 21-, and 28-day chemotherapy cycles to the rates of influenza infection before and after implementation of the recommendations. Citrix medication administration records and Meditech microbiology profiles were accessed to confirm flu vaccine administration and assess completed flu swab results. Patients were excluded from the study if they had weekly chemotherapy cycles, were receiving oral chemotherapy or did not have a documented influenza vaccination administration.

*Results:* Out of the 103 patient charts reviewed in this study, 47 patients in 2014 to 2015 and 56 patients in 2016 to 2017, no difference was found in the rates of influenza infection before and after implementation of the timing recommendations. 51% of patients in 2014 to 2015 and 27% of patients in 2016 to 2017 received their vaccinations within the recommended time frame. For all cycle lengths, the 2014 to 2015 group had higher rates of vaccinations administered within the recommended time period when compared to the 2016 to 2017 group. No patients in either group tested positive for influenza serotypes A and B. *Discussion:* No correlation was found in either group regarding flu vaccine and IV chemotherapy administration. While past studies suggested there may be significance in the timing of influenza vaccination in chemotherapy cycles, recent data has shown otherwise. The results from this retrospective review support recent data that the timing of vaccination may not influence the incidence of influenza infection.

*Recommendations:* (1) Completion of studies comparing influenza vaccine seroconversion rates to incidence of positive or negative flu swab results; (2) remove timing recommendations for influenza vaccinations for patients receiving intravenous chemotherapy.

## JL524. Pharmacy Financial Advocacy: Benefits of a Pharmacy Financial Advocate in the Oncology Clinic

Joe Weber, PharmD, BCOP, Lynnette Coolidge, PharmD, BCPS, BCOP, Kayla M. Hinsch, CPhT, and Kelly Carlson, RN, BS, MHA; Oncology Pharmacy, Sanford Cancer Center, Sioux Falls, South Dakota

*Background:* Comprehensive cancer care is often built on medical and psychosocial models. However, it is not uncommon for cancer programs to dismiss the importance of financial patient advocacy during the cancer journey. Increases in the number of new treatments approved by the Food and Drug Administration (FDA), along with established, complex oral and intravenous chemotherapy regimens, pose significant financial risk to both patients and the institutions that treat them. The numbers of patients unable to receive treatment due to financial constraints is increasing. There are several avenues available to help the patient successfully access chemotherapy and specialty medicines, however, an organization must be willing to commit resources to maximize the financial opportunities available. The purpose of this project was to decrease patient out-of-pocket expenses for oncology treatments through the development of a pharmacy financial advocacy program.

*Intervention:* This project was a process improvement project set at a tertiary care cancer center located in the Midwest. A dedicated financial advocacy program was established within the cancer center pharmacy, July 2014. The intent of the financial advocacy program was two-fold: (1) Develop expertise in the use of existing resources and (2) develop a process for seeking out new and alternative sources of funding. Dollars saved through co-pay assistance programs, free drug programs, and foundation assistance programs were tracked monthly.

*Results:* $91,000 in savings to patients and alternative revenue sources to the clinic was realized in the first month of the program. Within a year, the program transitioned from a shared role to a dedicated person maximizing the efficiencies. The pharmacy financial advocacy program has grown to an average savings of $423,000 monthly for all patient populations served within this program. The savings for FY17 totaled over $5,000,000.

*Conclusion:* Prior to the implementation of the pharmacy financial advocacy program, financial constraints were the main drivers in a patient’s decision as to whether he/she was going to proceed with treatment. As the role and program developed, so did the knowledge base and resources of the financial advocate. Utilizing foundations and financial assistance programs significantly decreased the patient’s out-of-pocket expenses and financial burden for chemotherapy treatments and other specialty medications.

*Implications:* A dedicated pharmacy financial advocate allows a comprehensive cancer center the opportunity to serve patients better, do the right thing, and limit financial risk to both the patients and the organization.

## JL525. Real-World Systemic Therapy Sequences Following a CDK 4/6 Inhibitor Regimen Among Post-Menopausal Women With Hormone Receptor–Positive Human Epidermal Growth Factor Receptor 2 Negative (HR+/HER2-) Metastatic Breast Cancer (mBC)

Derek Tang, PhD, BSPharm, Novartis Oncology, Nicole Princic, MS, Truven Health, Ayal Aizer, MD, Brigham and Women’s Hospital, Boston, Massachusetts, David Smith, PhD, Truven Health Analytics, William Johnson, MS, Truven Health Analytics, and Aditya Bardia, MD, Massachusetts General Hospital, Boston, Massachusetts

*Background:* While approval of CDK 4/6 inhibitors (CDK 4/6i), palbociclib and ribociclib, has changed the landscape of metastatic HR+/HER2– breast cancer, limited population-based data are available regarding therapy utilization for mBC patients after progression on CDK 4/6i. The aim of the current analysis is to describe real-world use of systemic therapies following a CDK 4/6i-based line (e.g., second line [2L], third line [3L], and fourth line [4L]) in the United States using a contemporary population-level data set.

*Methods:* Postmenopausal women with HR+/HER2– mBC were identified from MarketScan administrative claims databases between 1/1/2012 to 4/30/2017. The date of the first claim depicting metastatic disease was index. Patients had 12 months of continuous enrollment in their health plans prior to index and were followed until disenrollment, inpatient death, or study end (4/30/2017). Eligible mBC patients who received standard CDK 4/6i-based treatment were selected. A line of therapy regimen included all BC treatments during the first 45 days following initiation and ended at discontinuation (gap > 60 days and restart on new regimen), switch to new treatment, or censoring.

*Results:* 1,164 CDK 4/6i-based 1L, 2L, and 3L regimens followed by 375 subsequent post CDK 4/6i lines were included. Of these 42.4% were 2L (N = 159) and 57.6% (N = 216) were 3L/4L lines. Mean age at the start of first line was 62.7 years (SD = 10.5 years), 65.1% were commercially insured, and 65.1% of lines were completed during the study period (ended due to discontinuation or switching). Approximately one-third (37.7%) of 2L regimens (post-CDK 4/6i 1L) were endocrine only, 35.2% were chemotherapy-based, 15.1% were everolimus-based, 8.8% moved to a different CDK 4/6i-based regimen, and 3.1% were others. Across all post-CDK 4/6i lines (2L, 3L, 4L), most chemotherapy-based regimens were capecitabine- (48.1%) or paclitaxel-based regimens (24.4%); among the endocrine-only monotherapy regimens, the most prevalent sequence from the prior CDK 4/6i-based line of therapy was fulvestrant (39.7%) followed by letrozole (26.7%), and exemestane (14.5%).

*Conclusions:* After CDK 4/6i, patients transition to various regimens, including endocrine monotherapy, endocrine combination with everolimus, or chemotherapy-based regimens. Further research is needed to evaluate the relative efficacy and comparative effectiveness of these different regimens to identify optimal sequencing strategy in the 2nd-4thL setting for patients with mBC.

*Recommendations:* These data allow health-care providers and payers to understand real-world treatment sequencing following a CDK 4/6-based line of therapy and serve as a first step towards understanding the optimal sequencing tailored to patient’s clinical circumstances.

## JL526. Rituximab and Melanoma: A Case Report

Shimika Rasha, MD, MPH, Abhishek Nair, MD, and Zdenka Segota, MD; University of Miami, Miller School of Medicine, Holy Cross Hospital, Fort Lauderdale, Florida

*Background:* Rituximab is a monoclonal antibody directed against CD20 used in hematology for the treatment of non-Hodgkin lymphoma and chronic lymphocytic leukemia as well as in rheumatology to treat rheumatoid arthritis. Its use is also increasing in various inflammatory and autoimmune diseases. Thus, a large number of patients are being increasingly exposed to this medication. Its toxicity has been low, consisting mainly of immediate infusion reactions and infectious risks. To date, rituximab has not been associated with an increased risk of cancer. Several rituximab-associated melanoma cases, often at a metastatic stage, have been recently identified in various literature, the European pharmacovigilance database in 2012 and FDA report in 2017. This raises concern for melanoma onset secondary to rituximab in these immunosuppressed patients. Reviewing these cases also seems to raise the question of whether dermatological monitoring is essential in patients treated with rituximab, especially those who may have risk factors for melanoma.

*Discussion:* We report two cases of metastatic melanoma that occurred in two patients treated with rituximab for a prolonged period for non-Hodgkin lymphoma. Drawing conclusions on the harmful role of rituximab in melanoma occurrence or progression is still premature. Reporting these cases is essential to increase knowledge of the impact of rituximab on the incidence of secondary cancer and hence, to remain vigilant, especially as rituximab is currently been increasingly used and for a prolonged period. There are also ongoing studies interested in its use to treat melanoma at an early stage.

## JL527. Rural Infusion Centers Gaining Access to Expert Oncology and Infusion Support Through the Use of Telemedicine

Susan Halbritter, ANP-BC, AOCN®, Sanford Hematology and Oncology, Sioux Falls, South Dakota, Rachel Olson, MPA, Sanford Vermillion Medical Center, Vermillion, South Dakota, Jenna Kaiser, RN, BSN, OCN®, Sanford Hematology and Oncology, Sioux Falls, South Dakota, Janice McGuire, RN, MSN, Sanford Vermillion Medical Center, Vermillion, South Dakota, and Peggy Ann Dufek, RN, ADN, Douglas County Memorial Hospital, Armour, South Dakota

*Background:* Telemedicine has been described in the literature as an effective way to provide real-time expert medical care for patients residing in rural communities. The oncology literature is bereft in models that describe the use of telemedicine services to provide expert oncology support for rural infusion centers administering oncology therapies. Although many rural facilities have infusion centers that administer chemotherapy, those facilities do not have direct oversight by an oncologist or oncology-trained advanced practice professional (APP). Due to the high risk associated with chemotherapy agents and the potential for infusion reactions, oncologists have a limited list of agents that they will allow to be administered off-site. Thus, patients are often forced to travel to the tertiary care setting to receive their treatment. The purpose of this project was to determine if certified nurse practitioners (CNP) based in a tertiary care infusion center can provide oversight to two rural infusion centers through the use of telemedicine technology and the electronic medical record (EMR).

*Design:* This project, funded through an HRSA FORHP grant, was a descriptive, observational trial, set at a tertiary care center and at two rural infusion centers, located in the Upper Midwest. A dedicated oncology CNP within the tertiary care infusion center provides direct oversight to patient care; RNs provide rural infusion services. The intent of the virtual infusion project was to extend the CNP oversight into two rural infusion centers, using real-time support through the EMR and telemedicine capabilities.

*Results:* Over the course of a year, patient care was successfully transitioned from the tertiary infusion center to a site closer to home. The oversight of the CNP increased the comfort level of the prescribing oncologists. Complex treatments, previously only administered in the tertiary setting were safely transitioned into the rural setting. Patient and provider satisfaction surveys were overwhelmingly positive. Due to the success of the program, the project is in the process of adding an additional site.

*Conclusions:* The virtual infusion project demonstrated that an oncology CNP can provide safe and effective support to nurses in rural infusion centers through telemedicine technology. Telemedicine technology, the EMR, nursing education, shared policies, procedures and workflows allow for a seamless care-delivery model between the tertiary care setting and two rural infusion centers.

*Implications:* Rural communities provide oncology infusions, but have limited access to oncology providers. Real-time support through an oncology APP allows patients to receive complex treatments safely closer to home.

## JL528. Survivorship Care Planning in a Large, Multi-Clinic Cancer Program

Jamie Cairo, DNP, Aurora Cancer Care, and Dorry Mitchell, Aurora Health Care; Milwaukee, Wisconsin

*Background:* Aurora Health Care (AHC) is comprised of 15 hospitals and 22 oncology clinics. Aurora Cancer Care, a Commission on Cancer (CoC) accredited program, diagnoses nearly 8,000 new patients with cancer a year, more than any other health system in Wisconsin. The CoC’s Survivorship Standard 3.3 requires accredited cancer programs to provide curative intent cancer patients with survivorship counseling and a care plan. AHC was challenged to develop a model of survivorship care that can work at multiple sites across the system.

*Methods:* Workflow planning and education began at all oncology clinics in 2014. A system wide delivery plan was developed and launched in the first quarter of 2015. In 2016 the program set a goal of targeting 25% of eligible patients. Thirteen disease-specific survivorship care plans were built into the EMR with some auto population functionality. The care plans allow for multiple disciplines to enter data during or after the patient’s treatment. The model of survivorship care delivery is an "embedded consultation" with an advanced practice provider or cancer nurse navigator completing the care plan and meeting with the patient at the end of first-line treatment. A clarity report was launched Q3 of 2016.

*Results:* Projected volumes were estimated based on registry data from the previous year with a goal of disseminating 1,000 care plans in 2016 to meet the 25% CoC standard. Over 1,200 care plans were generated and/or were given to patients and their primary care providers. Breast, bone marrow transplant, prostate and colorectal cancer were the most used care plan templates.

*Conclusion:* Data review from 2016 demonstrates success with the current workflow and model of delivery. Aurora is on track to continue to meet the CoC’s yearly benchmarks. There has been a high level of engagement with the APPs and CNNs who have taken ownership of survivorship care planning, which has contributed to the success of the program thus far. The most significant barriers identified are the difficulty in identifying and tracking curative intent cancer patients to make sure that they are scheduled for a survivorship visit and developing a consistent reporting strategy using data from the EMR.

## JL529. The Advanced Practice Provider and Subcutaneous Rituximab: Implications for Practice

Jennifer Giarratana, MSN, ANP, AOCNP®, Joanne Henning, MSN, CRNP, APCNP, Nancy Driscoll, PA-C, Marlana Mattson, MSN, ACNP, AOCNP®, Kristien Miller, BSN, RN, Kristen Morris, BSN, RN, OCN®, and Sara Orndoff, MSN, OCN®, BMTCN; Genentech

*Background:* Rituximab (Rituxan), an anti-CD20 monoclonal antibody, was first approved in 1997 for treatment of follicular lymphoma (FL), diffuse large B-cell lymphoma (DLBCL) and chronic lymphocytic leukemia (CLL). Both oncology nurses and practices have over 2 decades of experience administering rituximab intravenously. The approximate 1.5 to 4 hours it takes to safely administer rituximab is a significant amount of time for both patients and oncology practices. Subcutaneous rituximab with recombinant human hyaluronidase (Rituxan Hycela), was approved by the FDA in June 2017 and allows for a subcutaneous administration of rituximab over 5 to 7 minutes. It is approved as a fixed dose; patients with FL or DLBCL will receive 1,400 mg and those with CLL will receive 1,600 mg.

*Intervention:* Advanced Practice Providers (APPs) play an integral role in oncology practices by providing a vital link to patients, the nursing team, and the oncologists. It is important for APPs to keep abreast of treatments that assist the oncology team in the management of patients. It is important that APPs understand the safety and efficacy data generated in the subcutaneous rituximab clinical development studies to enable the APP to educate the oncology team and patients on this new treatment option.

*Outcome Measures:* This poster will provide an overview of the clinical development program for subcutaneous rituximab. A summary of the pharmacokinetics, safety profile, efficacy results, and patient preference data will be provided. The mechanism of action, dosing regimens for indicated disease states and patient monitoring will be outlined. Decreased administration time, and fixed dose and single use vials, may improve accessibility to treatment and minimization of practice resources.

*Summary:* APPs have a pivotal role in the education and practice support during the integration of subcutaneous rituximab into the oncology practice. APPs’ assessment of the safety and efficacy data will enable them to provide education to the oncology staff and potential patients. APPs have a critical role in identifying appropriate patients and transitioning them to this new administration option of rituximab.

*Implications:* Subcutaneous rituximab provides an alternative route of administration to an IV infusion. This may improve the overall treatment experience for patients. Implications for practice include reduction in time for preparation and for administration, as well as simplification of the administration process and monitoring. The APPs’ role in transitioning patients and practices to this new treatment option will provide optimal and quantifiable benefits for both patient and practices.

## JL530. The Creation and Sustainability of an Effective After-Hours Coverage Model Utilizing Advanced Practice Providers

LaKeisha Day, PA-C, CMQ, Anayo Mbadugha, PA-C, and Todd Pickard, MMSc, PA-C, DFAAPA; The University of Texas MD Anderson Cancer Center, Houston, Texas

*Background:* Located in Houston’s Texas Medical Center, The University of Texas MD Anderson Cancer Center is a comprehensive cancer hospital that provides treatment at various stages of oncologic care. It is the largest oncology specialty hospital in the United States, with over 600 inpatient beds, 4 satellite locations around the city and over 25 affiliated entities within the US and internationally. With the exception of nursing staff, there were no full-time clinical staff members dedicated to after-hours patient care necessities. After-hours coverage for the entire hospital was provided by three oncology fellows and one medical resident, assigned to cover various oncology areas. This coverage model for such a large and complex patient population resulted in concerns for patient safety.

*Intervention:* In 2009, a 24-hour task force was formed to analyze the concerns and limitations of the current state of after-hour coverage and propose a way forward in providing after-hours coverage to suit the needs of the patients. The deliberations of the task force resulted in the creation of the Nocturnal Program. Launched February 8, 2011, with two full-time advanced practice providers (APPs) and a pool of 25 moonlighting physicians, the areas of most critical need were identified and coverage was assigned to the medical and surgical intensive care units, and one leukemia unit.

*Outcome:* By the end of 2011, the program consisted of 7 full-time APPs and 40 moonlighters, and has since grown into what is now one of the larger APP programs in the hospital, with 32 APPs and over 120 moonlighters. The hospital after-hours coverage expanded from seven providers to the current model of at least 21 providers every night of the week. As the hospital opened new coverage areas, the needs for patient care coverage were anticipated and the coverage models were adapted, including recently adding coverage for the pediatric transplant and intensive care units and the creation of an APP-run observation unit.

*Summary:* Although the creation of after-hours patient care models is not unique, the growth of the program in such a short time span can be attributed to a number of factors. The program was created independently from existing programs and utilized full-time APPs as first-line providers with moonlighting physicians serving as back-up. This team-based approach works because of the regulatory landscape in Texas, which due to generous collaboration and delegation laws, allows APPs to work with a high degree of autonomy.

## JL531. The Expanding Role of Surgical Physician Assistants in Clinical Oncology Research

Jenilette Velasco, MPAS, PA-C, and Steven H. Wei, MS, MPH, PA-C; The University of Texas MD Anderson Cancer Center, Houston, Texas

*Purpose:* To present an overview of the integration of surgical oncology advanced practice providers (APP) in clinical oncology research. Background: Recent changes in the surgery workforce have created a need for highly skilled and trained APPs to care for a complex patient population. In addition to various clinical roles, APPs are increasingly being utilized in non-clinical roles, including research. Surgical oncology, and in particular hepatico-pancreas-biliary (HPB), is a specialized discipline where APPs can contribute in a variety of ways to further enhance outcomes research, quality and performance improvement, as well as improve patient education. Furthermore, engaging in research roles beyond clinical care allow for increased collaboration and partnership between physicians and APPs. Involvement in different aspects of clinical research can also help the APP improve upon his or her clinical competencies, and lead to more opportunities in professional development, such as publishing and/or oral presentations. More studies are needed to evaluate the roles and impact of APPs in clinical research in the academic setting.

*Description:* We describe four specific roles that physician assistants (PA) are engaged in research in the outpatient, inpatient and surgical assist setting as part of a multi-step process in a complex HPB practice: (1) Screening patients for clinical or translational research trials; (2) Patient education and informed consent process; (3) Collection and evaluation of clinical data, including tissue and lab collection or analysis of data for outcomes research; (4) Implementing quality and performance improvement projects and identifying needs for future research. Objectives: (1) Discuss the expanding roles of APPs in the clinical oncology research setting; (2) Describe specific roles that APPs might engage in clinical research in a HPB surgical oncology practice; (3) Discuss potential benefits of APP involvement in oncology research beyond clinical care.

## JL532. The Quantity of Care Provided by Nurse Practitioners and Physician Assistants to Older Adults With Cancer

Lorinda A. Coombs, PhD(c), FNP-BC, AOCNP®, University of California San Francisco/Kaiser Permanente, San Francisco, California; Wendy Max, PhD, University of California San Francisco, San Francisco, California; Caroline Stephens, PhD, RN, GNP, University of California San Francisco, San Francisco, California, and Tatjana Kolevska, MD, University of California San Francisco/Kaiser Permanente, San Francisco, California

*Purposes/Aims:* The purpose of this study was to: (1) Describe the number of nurse practitioners (NPs) and physician assistants providing care to older adults with cancer and the amount of care provided in 2013 and (2) Identify and describe the specific types of malignancies for which NPs and PAs provide greater amounts of care.

*Background:* The leading cause of death for individuals age 40 to 79 years in the United States is cancer. Approximately 40% of all Americans will be diagnosed with a malignancy in their lifetime. Cancer is most frequently diagnosed in adults over 65 years of age, and the incidence is expected to dramatically increase between 2010 and 2050. Estimates of the oncology workforce suggest there may not be enough oncology physicians in practice to care for these older patients with cancer. The lack of a sufficient cancer workforce will directly impact patient care and represents a public health issue.

*Methods:* Utilizing 2013 data from the Surveillance, Epidemiology and End Result (SEER) program linked to Medicare enrollment and provider data, provide a description of the oncology workforce who provides care to Medicare patients with cancer. All members of the oncology workforce who provide cancer care to patients with Medicare are in the dataset, including NPs, PAs, and physician specialists. Patient malignancy was identified using the using the associated International Classification of Diseases (ICD) code. Analysis included calculation of proportions, percentiles within and among the provider groups to identify if there was a significant amount of care provided by NPs and PAs.

*Results:* A significant amount of ambulatory cancer care was provided by NPs and PAs, 6.9% of the workforce was NPs and PAs, compared with medical oncologists, hematologists or double boarded hematology/oncology physicians who comprised 9.5% of the workforce.

*Implications:* This is the first study to present non-self-reported data on cancer care provided by NPs and PAs to any population. The public health implications of this study include: identifying a solution to the growing need for cancer care and the anticipated cancer workforce deficit, possibly reducing Medicare workforce expenditures for cancer care by fully utilizing the current workforce, and allowing specialty physicians to focus on complex care while nurse practitioners provide care within their scope of practice. Partial research support provided by Alpha Eta Chapter UCSF Sigma Theta Tau International Honor Society of Nursing, Earle Anthony Social Sciences Fellowship and the American Academy of Nurse Practitioners.

## JL533. Utilizing the COMFORT Curriculum With Advanced Practice Providers to Enhance the Family Meeting Process and Improve Patient Care

Anne M. Kolenic, MSN, RN, AOCNS®, and Linda K. Baer, MSN, NP-C, AOCNP®; University Hospitals Seidman Cancer Center, Cleveland, Ohio

*Purpose:* To implement and evaluate an educational program for advanced practice providers (APP) utilizing the COMFORT Curriculum.

*Background:* The Institute of Medicine (IOM) has identified the need to address gaps in care of critically ill and dying as essential for ensuring a sustainable health care system. Strong communication skills have emerged from the IOM report as a central theme for improving care. Although cancer APPs are well positioned to hold goals of care (GOC) conversations and conduct family meetings, they often lack the communication skills, knowledge of, and confidence to do so. The COMFORT Communication Curriculum is a national health communication curriculum for oncology healthcare professionals. The aim of this project was to implement a training program focused on communication skills for GOC conversations and conducting family meetings.

*Intervention:* A two-part educational series was implemented by APPs trained in COMFORT curriculum. Based on findings from a learning needs assessment, the Communication, Family Caregivers, and Team modules were utilized. Each session was 50 minutes long and comprised of didactic information provided from COMFORT materials, case studies, and modeling of skills. The second session also provided a plan for standard documentation and billing options for GOC conversations. *Outcome*

*Measures:* The program evaluation assessed participation, knowledge, and confidence. Fifty-one APPs attended session one, while only 33 returned for part two due to clinical responsibilities. Overall, 54% of APPs attended both. Participants completed evaluations upon completion of each session. The initial outcomes reported were that 98% of participants responded they had gained knowledge and stated they would implement skills into clinical practice. Surveys will be sent at 2 and 6 weeks post training to assess intermediate outcomes for knowledge and confidence in performing skills, conducting family meetings, and engaging in GOC conversations. Results of these two time points will be available in September.

*Summary:* APPs responded positively to the educational content of both sessions and have anecdotally reported use of the skills in practice. Preliminary data has been shared with Senior Leaders who support dissemination of the education, tools, and documentation plan to interdisciplinary disease teams.

*Discussion:* Utilizing the COMFORT curriculum was feasible and enabled standardized content in communication and the process of family meetings to be presented to APPs. By educating these providers and enabling them with skills and tools to engage in GOC conversations, we anticipate greater transparency in the goals and wishes of our patients as they move within inpatient and outpatient settings.

